# Inhibition of the RAC/PAK Signaling Axis Enhances the Potency of MAPK Cascade Inhibitors Against Uveal Melanoma

**DOI:** 10.3390/biom15101425

**Published:** 2025-10-07

**Authors:** Alexei A. Maslov, Nicholas H. Trageser, Julia V. Kichina, Haya Elamir, Evelyn Gardner, Frances Teaman, Vera Vishwanath, Scott M. Dugas, Johanna Heid, Alexander Y. Maslov, Henry G. Withers, Anna Bianchi-Smiraglia, Katerina I. Leonova, Mikhail A. Nikiforov, Eugene S. Kandel

**Affiliations:** 1 Department of Cell Stress Biology, Roswell Park Comprehensive Cancer Center, Buffalo, NY 14263, USA; 2 Department of Immunology, Roswell Park Comprehensive Cancer Center, Buffalo, NY 14263, USA; 3 Perlmutter Cancer Center at NYU Langone Health, New York, NY 10016, USA; 4 Department of Genetics, Albert Einstein College of Medicine, Bronx, NY 10461, USA; 5 Department of Biostatistics and Bioinformatics, Roswell Park Comprehensive Cancer Center, Buffalo, NY 14263, USA; 6 Department of Pathology, Duke University, Durham, NC 27710, USA

**Keywords:** targeted therapy, uveal melanoma, PAK1, IMPDH

## Abstract

Uveal melanoma is a melanocyte-derived malignancy of the eye with a high propensity for liver metastasis. Metastatic uveal melanoma is associated with high mortality and is poorly responsive to currently available therapies. Most uveal melanoma cases are driven by activating mutations in GNAQ and GNA11 genes, which convey oncogenic signaling through the mitogen-activated protein kinase (MAPK) pathway. Despite promising early results, safe doses of pharmacological inhibitors of the MAPK cascade failed to effectively control uveal melanoma in human trials. Considering the role of the RAC/PAK signaling axis as a co-regulator of the MAPK cascade, we set forth to investigate whether the efficacy of MAPK cascade inhibitors in pre-clinical models may be enhanced by direct inhibition of RAC and PAK proteins, or by indirect control of RAC via inhibition of guanylate biosynthesis. We observed that pharmacological inhibition of RAC, PAK and the key guanylate biosynthesis enzyme IMPDH significantly synergized with various inhibitors of the MAPK cascade in suppressing oncogenic signaling and the growth of uveal melanoma cells. In a mouse model, the addition of an IMPDH inhibitor to the treatment regimen significantly enhanced the ability of a MAPK cascade inhibitor to improve the survival of tumor-bearing animals. Targeting of the RAC/PAK axis provides a new strategy to increase the efficacy of targeted therapies in uveal melanoma. While RAC and PAK inhibitors are still undergoing pre-clinical development, clinically available inhibitors of IMPDH offer an opportunity to test the efficacy of this novel synergistic combination in the context of human disease.

## 1. Introduction

Uveal melanoma (UM) is the most common intraocular malignancy in adults. It is derived from melanocytes, but is significantly different from the more common melanoma of the skin in terms of the underlying molecular pathology, disease trajectory and therapeutic options [1]. Multiple treatment modalities exist for the management of primary UM. While surgical enucleation remains an option, eye-sparing localized treatments include brachytherapy, transpupillary thermotherapy, photodynamic therapy, proton beam therapy, etc. [2,3] These techniques afford a relatively effective control of the localized tumor. Indeed, survival among Stage 1 patients is well over 90% [4]. Unfortunately, about half of patients develop metastasis, with the liver being the predominant site of secondary growth [5,6], and the treatment options for metastatic UM are extremely limited [1,4]. Systemic treatments for metastatic UM include chemotherapy, targeted therapies, and immunotherapies, although their efficacy has generally been very limited [1,4,7]. Until recently, the quest for a cure for metastatic UM has been described as a “long history of disappointments” [8]. Some encouraging results were obtained with the use of immune checkpoint inhibitors (ICIs), such as ipilimumab (anti-CTLA4) and nivolumab (anti-PD-1). While ICI are largely ineffective as single agents, ICI-based combination regimens showed some benefits in early clinical trials, albeit with considerable toxicity [4]. Furthermore, a definitive benefit was obtained with the ImmTAC (Immune mobilizing monoclonal T-cell receptor Against Cancer) tebentafusp [9]. Tebentafusp targets the melanoma-specific antigen gp100 presented by HLA on the cell surface [10] and provides a statistically significant, but modest (21.7 vs. 16.0 months), improvement in median overall survival [9]. Unfortunately, the use of tebentafusp is applicable only to approximately 50% of the UM patients who carry the HLA-A*02:01 allele, and even in that category, it achieves an overall response rate of only 9% [9]. Thus, the problem of effective management of metastatic UM remains largely unsolved.

A better understanding of the molecular pathology underlying the initiation and progression of UM may offer actionable insights into the management of the disease. It is widely recognized that, unlike cutaneous melanoma, the vast majority (>90%) of UM cases are driven by activating mutations in the GNAQ and GNA11 genes, which encode the alpha subunits of heterotrimeric G-proteins [7]. Under normal physiological conditions, GNAQ and GNA11 proteins are involved in G-protein-coupled receptor (GPCR) signaling as alpha-subunits of heterotrimeric G-proteins [11]. Indeed, constitutive activation of the GPCR CYSLTR2, which also occurs in a subset of UM, is believed to be functionally similar to GNAQ/GNA11 activation [7]. Constitutive signaling by GNAQ and GNA11 stimulates a number of oncogenic pathways, including PI3K/AKT, Hippo/YAP and the MAP kinase cascade [12]. The critical dependence of UM cells on the latter [13] made it an especially attractive target for therapeutic intervention. Despite some promising earlier observations [14], the phase III trial of a MEK inhibitor (MEKi) selumetinib found no survival benefit at tolerable drug doses [15], underscoring the need for more effective MEK inhibition while maintaining drug safety [15]. Several other early-phase attempts to exploit MEKi in UM care also failed to yield encouraging results [16,17]. This unfortunate situation brings up the question of whether the efficacy of MEKi can be enhanced by combining it with a synergizing agent, so that the combination becomes efficacious even in cases when MEKi alone is essentially ineffectual. This approach is analogous to that proposed for similarly resistant RAS-driven malignancies [18]. Prior research in other malignancies suggested that the inhibition of the MAPK cascade can be augmented by concomitant inhibition of PAK1 (p21-activated kinase 1) [19,20,21], an enzyme that has been implicated in co-stimulation of both CRAF and MEK1 [22,23,24,25] as well as in a variety of other oncogenic phenomena [23,24]. PAK1 and its eponymous activator RAC1 (also known as p21^RAC1^) are important components of signaling by oncogenic RAS proteins and represent a targetable vulnerability in cancer cells driven by RAS oncogenes [20,26,27,28,29]. Importantly, there is mounting evidence of RAS and RAC1 involvement downstream of GNAQ/GNA11 and GPCRs [30,31,32]. Furthermore, PAK1 activity is reportedly elevated in UM, where it, along with PAK4, contributes to the invasiveness of the cancer cells [33]. We therefore hypothesized that inhibition of the RAC/PAK signaling axis, particularly in combination with MAPK cascade inhibition, could be beneficial for the management of UM [34].

## 2. Materials and Methods

### 2.1. Cell Culture and Reagents

All cell lines were cultured inside humidified incubators that were maintained at 37 °C and 5% CO_2_. The uveal melanoma cell lines Mel202 [35] and Mel270 [35] (gifts from Dr. W. Harbour), 92.1 [36] (a gift from Dr. H. Grossniklaus) and MP41 (purchased from ATCC) were extensively characterized previously [37,38]. These cell lines were maintained in Roswell Park Memorial Institute (RPMI) 1640 Medium (Thermo Fisher Scientific, Waltham, MA, USA) containing penicillin (100 Units/mL), streptomycin (100 μg/mL), and 10% FBS. The medium was additionally supplemented with MEM Non-Essential Amino Acids Solution (Thermo Fisher Scientific), MEM Vitamin Solution (Thermo Fisher Scientific), and Sodium Pyruvate (Thermo Fisher Scientific). WI-38cells [39] immortalized by expression of hTERT [40] were a gift of Dr. A. Gudkov and cultured in high-glucose Dulbecco’s Modified Eagle Medium (DMEM; Thermo Fisher Scientific) containing penicillin (100 Units/mL), streptomycin (100 ug/mL) and 10% FBS All cell lines were tested for mycoplasma using the MycoAlert Mycoplasma Detection Kit (Lonza Bioscience, Walkersville, MD, USA).

Selumetinib was purchased from Selleckchem, Houston, TX, USA (S1008), MedChemExpress, Monmouth Junction, NJ, USA (HY-50706) and Adooq, Irvine, CA, USA (10257-250). Cobimetinib was purchased from Adooq (#A11441). Mirdametinib was purchased from Selleckchem (S1036). Binimetinib was purchased from Adooq (A11493). Ribavirin was purchased from Selleckchem (S2504), MedChemExpress (HY-B0434) and Adooq (A10788). Mizoribine was purchased from Selleckchem (S1384) and Cayman Chemical, Ann Arbor, MI, USA (23128). IPA3 was purchased from Selleckchem (S7093) and Cayman Chemical (4759). PF3758309 was purchased from Selleckchem (S7904). EHT1864 was purchased from Adooq (A13886). No differences in efficacy were noted whenever the same compound was procured from multiple vendors.

### 2.2. Western Blotting and Antibodies

Cells were lysed in 1x RIPA Lysis Buffer (EMD Millipore, Darmstadt, Germany) supplemented with PhosSTOP Phosphatase Inhibitor and Complete Protease Inhibitor cocktails (Roche, Basel, Switzerland). pERK (sc-7383), total ERK (sc-514302), HRP-conjugated β-actin (sc-47778) primary antibodies and mouse anti-rabbit IgG HRP secondary antibody (sc-2357) were all purchased from Santa Cruz Biotechnology, Dallas, TX, USA. IMPDH2 primary antibody (12948-AP) was purchased from Proteintech, Rosemont, IL, USA. All prepared protein samples were separated on Mini-PROTEAN TGX gels (Bio-Rad, Hercules, CA, USA) in denaturing conditions. Following protein transfer onto PVDF membranes (Thermo Fisher Scientific), the membranes were blocked in either 5% Blotting Grade Blocker Non-Fat Dry Milk (Bio-Rad) dissolved in Tris-Borate-SDS-Tween 20 (TBST) or 5% BSA-TBST per the recommended probing conditions for each antibody followed by overnight probing at 4 °C. Following incubation with primary antibody, membranes were washed in TBST and incubated in secondary antibody diluted in 5%–Blotting Grade Blocker Non-Fat Dry Milk-TBST for 1 h at room temperature on a shaker. Immunobilon Classico (EMD Millipore) and SuperSignal West Pico PLUS Chemiluminescent Substrate (Thermo Fisher Scientific) were used for developing membranes for chemiluminescent imaging. The immunnoblots were imaged on a Bio-Rad ChemiDoc Touch Imaging System (software version 1.2.0.12) and the intensities of individual bands were quantified using Fiji software (ImageJ version 1.54P). The results were compared in Microsoft Excel using the TTEST function of the Analysis ToolPak add-in.

### 2.3. Rac1 Activity Assay

Rac1 activity was measured in protein lysates collected from cell lines, which were treated under various conditions, using the Active GTPase Kit (Cell Signaling Technology, Danvers, MA, USA; 1860S), which uses a PAK1-derived bait. Lysates were prepared per the kit’s instructions (the 1 × Lysis/Binding/Wash buffer was supplemented with Complete Protease Inhibitor Cocktail). Samples containing precipitated active-Rac1 and the corresponding input samples were immunoblotted for Rac1 using the primary antibody supplied with the kit. The signals were acquired using Bio-Rad ChemiDoc Touch Imaging System and quantified using Fiji software (ImageJ version 1.54P).

### 2.4. Cell Number Comparison in Treated Cultures

Compounds were added to sub-confluent cultures of cells that were plated the night before. Following the desired length of treatment, the number of remaining cells was compared using the methylene blue staining and extraction method as described [41] using a PerkinElmer Victor X3 Multimode Plate Reader, PerkinElmer, Inc., Shelton, CT, USA. Background-corrected values for each treatment condition were normalized to the average values obtained in parallel cultures of cells treated with a corresponding vehicle control.

### 2.5. EdU-Incorporation Assay

The cells were plated as sub-confluent cultures overnight onto 8-well chambered microscope slides and treated for the desired length of time under the desired treatment conditions. Following treatment, cells were pulsed with 5uM EdU for 1 h before being fixed in 3.7% formaldehyde and permeabilized in 0.5% Triton X-100 solution. Subsequently, the cells were stained with fluorescent azide AF 594 using the Click-&-Go^TM^ Edu Cell Proliferation Kit for Imaging (Click Chemistry Tools) for 30 min at room temperature, followed by 5 μg/mL Hoechst 33342 for 30 min at room temperature. Stained cells were imaged using the EVOS M5000 (Thermo Fisher Scientific) fluorescent microscope with integrated camera and imaging software. Captured images were analyzed using Fiji software [42] with ImageJ version 1.54P.

### 2.6. Quantitative Reverse Transcription PCR (qRT-PCR)

Overnight cultures of 92.1 cells were treated with 10 uM ribavirin and 30 nM selumetinib, alone or in combination, or with DMSO as a vehicle control. Following a 72 h treatment, RNA was extracted using the *Quick*-RNA Miniprep Kit (Zymo Research, Irvine, CA, USA) following the manufacturer’s instructions. The RNA was reverse-transcribed using qScript Reverse Transcriptase kit (Quantabio, Beverly, MA, USA ), and quantitative real-time PCR was performed with iTaq Universal Probes Supermix (Bio-Rad, Hercules, USA) in CFX96 Real-time System (Bio-Rad) using assays Hs02786624_g1 for GAPDH and Hs00152928_m1 for EGR1 (both–Thermo Fisher Scientific) following manufacturers’ instructions. The “ΔΔC_t_” method [43] was used to calculate relative fold-change in gene expression among the samples.

### 2.7. Animal Experimentation

The animal work was performed in accordance with the protocol approved by the Institutional Animal Care and Use Committee of Roswell Park Comprehensive Cancer Center under protocol 1462 (originally approved 21 December 2021). Xenografts were established by injecting 2 × 10^6^ cells subcutaneously into the right flanks of female SCID mice. Tumor volumes were calculated using the formula (L × W^2^)/2 [44]. Established tumors were allowed to grow until they reached approximately 100 mm^3^, after which animals were randomly assigned to each treatment group and received oral gavage daily in 100 uL. The animals were sacrificed when they became moribund, or their tumor volume exceeded 1.5 cm^3^.

### 2.8. Comparison of Intracellular GTP Content

Nucleotides were extracted and analyzed essentially as previously described [45,46]. Briefly, 5 × 10^6^ cells were collected by trypsinization after the indicated treatments and extracted in 0.4 N perchloric acid, followed by neutralization to pH 8 with potassium hydroxide. NTPs were separated using a strong anion exchange reverse phase column (Millipore Sigma, Burlington, VT, USA; Cat # 50193-U) and a gradient high-performance liquid chromatography system (Agilent 1100, Santa Clara, CA, USA) equipped with a photodiode array detector and controlled by the Agilent ChemStation B.04.03-SP1 software. Elution in a linear gradient of ammonium phosphate buffers occurred over 30 min. NTPs were identified by their UV absorbance spectrum and quantified by comparing the integrated area of their absorbance peaks to that of known NTPs standards.

### 2.9. Statistical Methods

For in vitro cell survival assays, drug synergy and the corresponding *p*-values were calculated according to the Bliss synergy model using the SynergyFinder software (version# 16.01.2024-R-3.8.2-dev) [47]. Gene Expression Interactive Expression Profiling Analysis tool [48] was used to plot the disease-free survival curves of uveal melanoma patients. GraphPad Prism 10 was used for Student’s *t*-test, ANOVA, IC50 calculations and Kaplan–Meier survival analysis. *p*-values below 0.05 were used as an indication of statistical significance.

## 3. Results

### 3.1. GNAQ- and GNA11-Mutant Uveal Melanomas Are Vulnerable to PAK Inhibition

The MAPK cascade, initiated by common activating mutations in GNAQ and GNA11 [49,50], is the main oncogenic pathway driving uveal melanoma [13]. GNAQ and GNA11 are components of GPCR signaling [51], which relies on downstream small GTPases, such as RAC1, to relay proliferative signals [32]. The oncogenic activity of GNAQ/GNA11 is also dependent on the activation of Ras [30,31]. Importantly, in multiple model systems, the Ras-driven pathway of oncogenic transformation was shown to depend on the function of RAC1 and its immediate downstream effector PAK1 [26,27,29,52,53], representing a potentially exploitable vulnerability in a subset of cancers [20,54]. Therefore, we set forth to investigate whether GNAQ/GNA11-driven uveal melanoma cells also exhibit heightened sensitivity to PAK inhibition.

**Figure 1:**
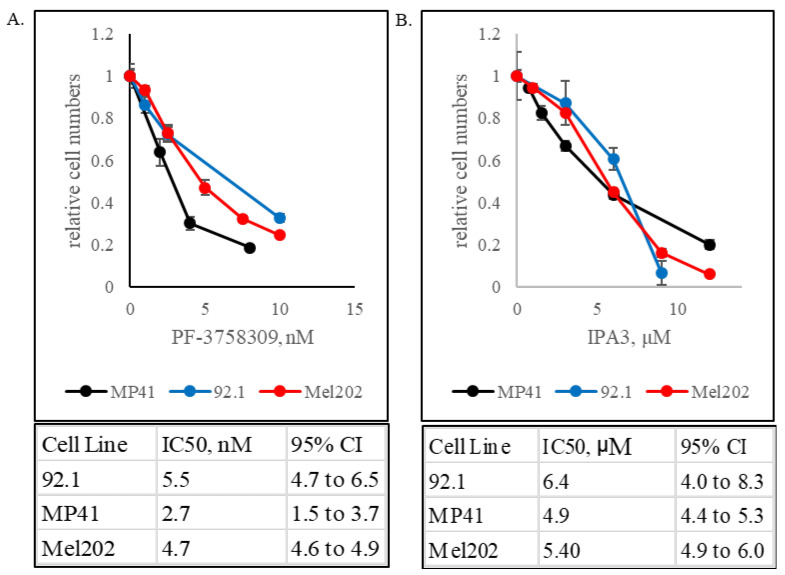
GNAQ- and GNA11-mutant uveal melanoma cells are sensitive to PAK inhibitors. (**A**). The indicated concentrations of a pan-PAK inhibitor PF3758309 were added to the cultures of 92.1, Mel202 and MP41 uveal melanoma cells. Five days later, cell numbers were compared using the methylene blue staining and extraction method. The values for each cell line were normalized to those in the respective control cultures. Error bars denote standard deviations of quadruplicates. (**B**). 92.1, Mel202 and MP41 uveal melanoma cells were treated with a group I PAK inhibitor IPA3 and the results were analyzed as in A. Half-maximal inhibitory concentrations (IC50) with corresponding 95% confidence intervals (95% CI) are shown.

To this end, we treated uveal melanoma cell lines 92.1 (GNAQ mutant), Mel202 (GNAQ mutant), and MP41 (GNA11 mutant) with a range of doses of IPA3 and PF3758309. IPA3 is specific against group I PAKs [55], while PF3758309 is a pan-PAK inhibitor [56]. The cell lines showed profound sensitivity to both inhibitors (Figure 1). Importantly, the drug concentrations used in these experiments are known to be effective against cancer cells that are driven by RAS, but not BRAF oncogenes [20,57]. Furthermore, they are protective for non-transformed cells under certain stress conditions [58]. Our observation supports the hypothesis about the PAK-dependence of uveal melanoma. Furthermore, given that IPA3 and PF3758309 have differing mechanisms of action and only partially overlapping specificities, one may predict that it is the group I PAKs that are vitally important to GNAQ- and GNA11-mutant cells.

### 3.2. Inhibitors of the RAC/PAK Signaling Axis Synergize with the MEK Inhibitors in Uveal Melanoma Cells

**Figure 2:**
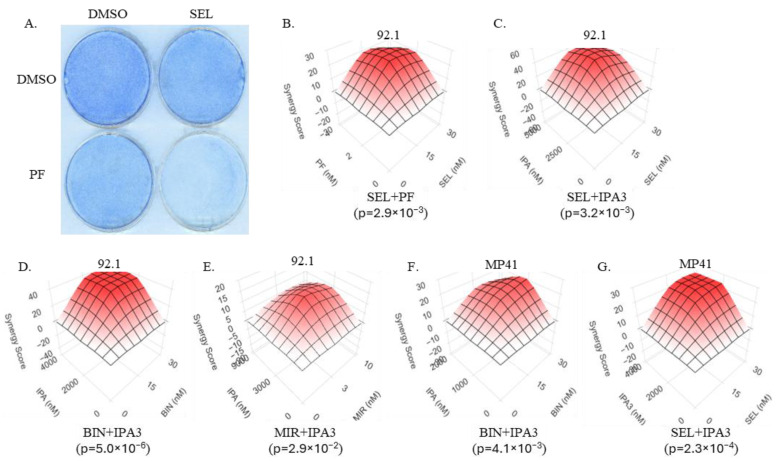
MEK inhibitors and PAK inhibitors synergistically suppress uveal melanoma cells. (**A**). 92.1 cells treated with MEKi selumetinib (30 nM), PAKi PF3758309 (4 nM), or the corresponding vehicle (DMSO) control were fixed and visualized by methylene blue staining. (**B**–**G**). Bliss synergy scores for various MEKi/PAKi combinations in uveal melanoma cell lines. (**B**). 92.1 treated with MEKi selumetinib and PAKi PF3758309. (**C**). 92.1 treated with MEKi selumetinib and PAKi IPA3. (**D**). 92.1 treated with MEKi binimetinib and PAKi IPA3. (**E**). 92.1 treated with MEKi mirdametinib and PAKi IPA3. (**F**). MP41 treated with MEKi binimetinib and PAKi IPA3. (**G**). MP41 treated with MEKi selumetinib and PAKi IPA3. All cultures were analyzed 5 days after the addition of the indicated compounds. Relative survival in (**B**–**G**) was measured using methylene blue staining and extraction method. Each condition was tested in quadruplicate. SynergyFinder was used to calculate Bliss synergy scores with *p*-values for each experiment. “SEL”—selumetinib, “BIN”—binimetinib, “MIR”—mirdametinib, “PF”—PF3758309, “IPA”—IPA3.

Despite the reliance of uveal melanomas on the MAPK cascade, the inhibition of this pathway with clinically achievable doses of MEK inhibitors, alone or in combination with chemotherapy, fail to achieve a significant improvement in patient overall survival [14,15,17,59]. Given that PAK inhibition sensitizes mutant RAS- and BRAF-driven cell lines to the inhibition of the MAPK cascade [19,20,21], we hypothesized that PAK inhibition would also be able to sensitize GNAQ and GNA11 mutant uveal melanoma cells to MEK inhibitors. We used uveal melanoma cell lines and a variety of experimental conditions to test the synergy between different PAK inhibitors (PAKi) and several different MEK inhibitors (MEKi) (Figure 2).

**Figure 3:**
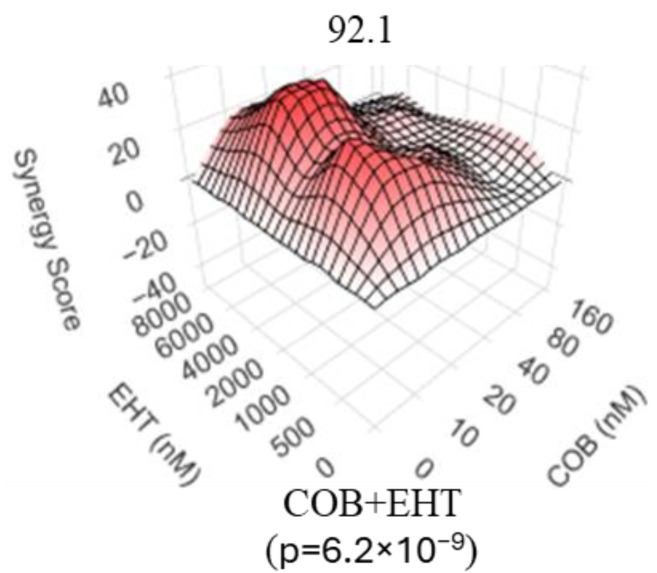
Rac inhibitor synergizes with MEK inhibition in uveal melanoma cells. 92.1 cells were analyzed 4 days after the addition of various combinations of EHT1864 (RACi) and cobimetinib (MEKi). Each condition was tested in quadruplicate. Relative cell numbers were compared using methylene blue staining and extraction method. SynergyFinder was used to calculate the Bliss synergy score with the corresponding *p*-value. “EHT”—EHT1864, “COB”—cobimetinib.

Considering the role of RAC1 as both the eponymous activator of PAK proteins and an essential element of GNAQ/GNA11-initiated oncogenic signaling, we predicted that a RAC inhibitor, akin to PAK inhibitors, would synergize with an inhibitor of the MAPK cascade. Indeed, we observed that a RAC inhibitor EHT1864 [60] synergizes with the MEK inhibitor cobimetinib in suppressing UM cells (Figure 3).

Taken together, our observations suggest that therapeutic targeting of the RAC/PAK signaling axis in the context of uveal melanoma may be a viable strategy to improve the efficacy of MEK inhibitors.

### 3.3. Inhibitors of GTP Biosynthesis Synergize with MEK Inhibitors in Uveal Melanoma Cells

While the role of PAK signaling as a promising therapeutic target in cancer is long-recognized [34,61], there are currently no clinically available inhibitors of this enzyme [34]. Interestingly, Rac1 activity is hypersensitive even to moderate changes in intracellular GTP levels [62]. Indeed, in the context of cutaneous melanoma, reduced GTP biosynthesis is known to attenuate some of the transformation-associated phenotypes in a RAC1-dependent manner [62]. RAC1 activity is supported by high local concentration of GTP, which is achieved by a direct association between RAC1 and GTP biosynthesis enzymes, such as IMPDH2 [63]. Indeed, inhibition of IMPDH2 reduces the activity of RAC1, as evidenced by its reduced binding to PAK1 [63]. Accordingly, IMPDH inhibitors, such as ribavirin and mizoribine, are able to reduce both the levels of GTP and the levels of active RAC1 in treated cells (Figures S1 and S2). Importantly, a number of IMPDH inhibitors are currently used in medical practice worldwide, making them attractive candidates for re-purposing for new applications. We hypothesized that, akin to PAK and RAC inhibitors, inhibitors of IMPDH would synergize with inhibitors of MEK in suppressing GNAQ- and GNA11-driven cancer cells.

**Figure 4:**
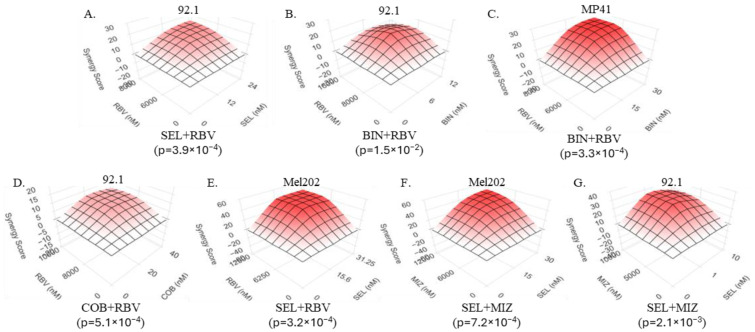
IMPDH inhibitors synergize with MEK inhibitors in suppressing uveal melanoma cells In Vitro. (**A**). 92.1 treated with selumetinib and RBV. (**B**). 92.1 treated with binimetinib and RBV. (**C**). MP41 treated with binimetinib and RBV. (**D**). 92.1 treated with cobimetinib and RBV. (**E**). Mel202 treated with selumetinib and RBV. (**F**). Mel202 treated with mizoribine and selumetinib. (**G**). 92.1 treated with selumetinib and mizoribine. All cultures were analyzed 5 days after the addition of the indicated compounds. Relative survival was measured using methylene blue staining and extraction method. Each condition was tested in quadruplicate. SynergyFinder was used to calculate Bliss synergy with the indicated *p*-values for each experiment. “SEL”—selumetinib, “BIN”—binimetinib, “COB”—cobimetinib, “RBV”—ribavirin, “MIZ”—mizoribine.

To test this prediction, we treated different uveal melanoma cell lines with various combinations of IMPDH and MEK inhibitors. The results revealed significant cooperativity between the two families of compounds (Figure 4A–G). Importantly, the IMPDH inhibitors used (ribavirin and mizoribine) share the target, but differ in their chemical properties and mechanisms of action [64,65], as do the inhibitors of MEK [66], suggesting that the synergy phenomenon is reflective of the on-target activities of these molecules.

Interestingly, the doses of MEKi and IMPDHi, which were synergistically toxic to uveal melanoma cells, were well tolerated by non-transformed human cells (Figure S3), suggesting cancer-specificity of the treatment.

### 3.4. Enhanced Suppression of the MAPK Signaling Cascade by Combined Treatments

**Figure 5:**
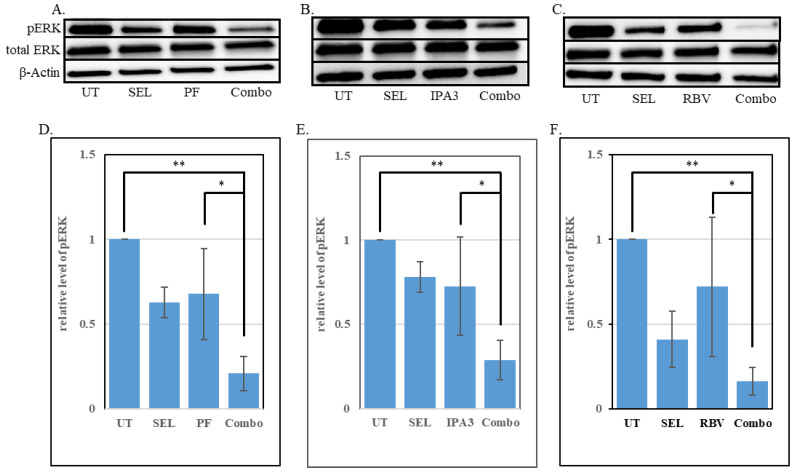
Inhibitors of PAK and IMPDH augment MEK inhibitor selumetinib in suppressing the signaling of the MAPK cascade. (**A**). Lysates of 92.1 cells treated for 72 h with selumetinib (30 nM; “SEL”), PF3758309 (4 nM; “PF”) or their combination (“Combo”) were compared to those of untreated control cultures (“UT”; exposed to the vehicle only) by probing with the antibodies for phosphorylated ERK (top), total ERK (middle) and β-actin (bottom). (**B**). Lysates of 92.1 cells treated for 72 h with selumetinib (30 nM; “SEL”), IPA3 (4 µM), or their combination (“Combo”) were analyzed as in A. (**C**). Lysates of 92.1 cells treated for 72 h with selumetinib (30 nM; “SEL”), ribavirin (10 µM; “RBV”) or their combination (“Combo”) were analyzed as in A. (**D**). Three independent experiments performed as in A were analyzed quantitatively, and the intensity of the pERK band was normalized to that of β-actin in each sample. The resulting values from each experiment were further normalized to those in parallel untreated controls. The bars represent averages and the error bars—standard deviations of the three experiments. (**E**). Three independent experiments were performed as in B and analyzed as in D. (**F**). Three independent experiments were performed as in C and analyzed as in D. “*” denotes *p* < 0.05. “**” denotes *p* < 0.01. The original Western Blotting images are in the Supplementary Materials.

The efficacy of MAPK cascade inhibitors is often reflected in their ability to lower the levels of active (phosphorylated) ERKs [67]. For example, in colon cancer, it has been observed that the clinical efficacy of combined EGFR/BRAF/MEK targeting correlated with increased ERK suppression [68]. Furthermore, among the biopsies of cutaneous melanomas taken from patients treated with BRAFi, greater reduction in phosphorylated ERKs correlated with favorable clinical responses [69]. The compounds that enhance the potency of MAPK cascade inhibitors In Vitro often also reduce ERK phosphorylation [21,70]. Accordingly, we observed that the addition of PAK inhibitors PF3758309 and IPA3 significantly increases the ability of a MEK inhibitor, selumetinib, to reduce the levels of phopho-ERK in 92.1 cells. The abundance of phospho-ERK in combination-treated cells was lower than that in the untreated cells and in the ones treated with the MEKi alone (Figure 5A,B). Once again, IMPDH inhibitor ribavirin acted similarly to PAK inhibitors by enahncing the effect of selumetinib (Figure 5C). This phenomenon was also seen in Mel202 cells (Figure S4).

**Figure 6:**
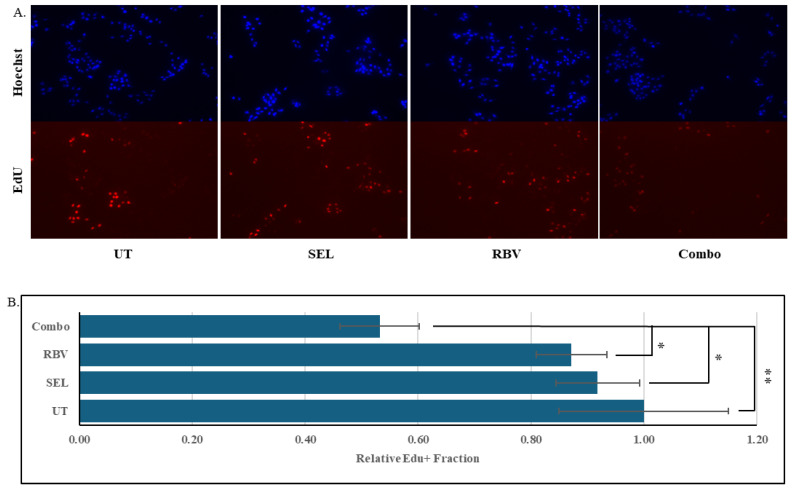
MEKi and IMPDHi synergistically suppress proliferation of uveal melanoma cells In Vitro. (**A**). 30 nM selumetinib and 10 μM ribavirin, individually or in combination, were added to the cultures of 92.1 cells. 48 h later, the cells were pulsed with 5 μM EdU for 1 h. The cells were fixed and stained for the incorporated EdU using the Edu AF 594 Imaging Kit, DNA was stained using Hoechst 33342 dye, and the signals were visualized by fluorescent microscopy. (**B**). For each culture treated as in A, the fractions of EdU-positive cells were determined by analyzing randomly chosen fields using Fiji software (ImageJ version 1.54P), and the values are shown relative to those among the untreated cells. Error bars denote standard deviations. “*” denotes *p* < 0.05. “**” denotes *p* < 0.01 (Student’s *t*-test).

Activation of the MAPK cascade is the major driver of cell proliferation in various cancers [66,71], and its effective suppression is often characterized by the reduced ability of cells to enter the DNA synthesis phase of the cell cycle [70]. We investigated the incorporation of a thymidine analogue 5-Ethynyl-2′-deoxyuridine (EdU) [72] into the DNA of uveal melanoma cells treated with selumetinib, ribavirin, or a combination thereof. Notably, the combination was able to reduce DNA synthesis even when the individual drugs were essentially ineffectual (Figure 6).

Overall, the data indicate that the control of proliferation and oncogenic signaling in uveal melanoma by MEK inhibitors could be significantly improved by the addition of IMPDH inhibitors.

### 3.5. An IMPDHi and MEKi Combination Reduces the Expression of EGR1 in Uveal Melanoma Cells

**Figure 7:**
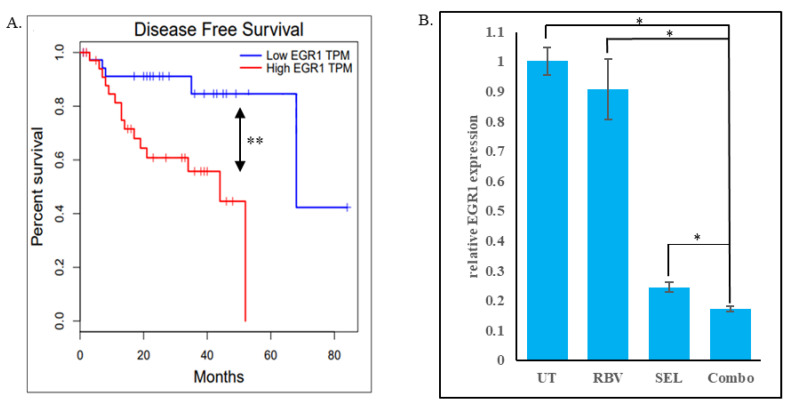
A combination of MEK and IMPDH inhibitors improves the suppression of the marker of uveal melanoma progression ERG1. (**A**). Kaplan–Meier analysis comparing the disease-free survival of uveal melanoma patients in with high or low EGR1 expression (split along the median). The hazard ratio between the groups is 4.7. “**”—*p* = 0.006. (**B**). The expression of ERG1 mRNA in 92.1 cells treated for 72 h with 30 nM selumetinib (“SEL”), 10µM ribavirin (“RBV”), selumetinib and ribavirin together (“Combo”), or untreated (“UT”) was compared by quantitative RT-PCR using GAPDH as an internal control. Bars represent EGR1 mRNA expression relative to that in the untreated cells. Error bars display the standard errors of the mean from biological triplicates. “*”—*p* < 0.05 (Student’s *t*-test with Bonferroni correction).

*EGR1* (Early Growth Response 1) is a transcription factor that regulates the expression of various genes involved in cell growth, differentiation, and survival, playing a significant role in cancer development and progression [73]. EGR1 gene is a well-established transcriptional target of the MAPK cascade [73]. It was reported that EGR1 expression decreases in response to MEK inhibitor treatment in several melanoma cell lines and that knockdown of EGR1 causes a reduction in viability [74]. In uveal melanoma cells in particular, treatment with a MEK inhibitor results in a potent downregulation of both EGR1 and its transcriptional targets [75], while forced reduction in EGR1 expression was reported to attenuate growth and impart sensitivity to MEK inhibition [76]. Molecular analysis of uveal melanomas revealed that not only does EGR1 promote angiogenesis and tumor growth [77], but its expression is also strongly associated with liver metastasis [78]. Accordingly, lower expression of EGR1 is associated with significantly prolonged disease-free survival among uveal melanoma patients (Figure 7A).

Considering the roles of EGR1 as an indicator of the MAPK cascade activity, a determinant of MEKi efficacy, and a correlate of clinical outcomes in uveal melanoma, we explored whether the addition of IMPDHi to a MEKi enhances the suppression of EGR1 expression. To this end, 92.1 cells were treated for 72 h with selumetinib, ribavirin, or a combination of both drugs, and the fold reduction in EGR1 mRNA level relative to that in the untreated cells was determined using quantitative RT-PCR. The results indicate that, as expected, the MEK inhibitor selumetinib reduced the expression of EGR1, and this effect was significantly boosted when selumetinib was combined with ribavirin (Figure 7B). The enhanced inhibition of EGR1 transcription by the drug combination is consistent with the improved suppression of the MAPK cascade. It is also encouraging for potential applications of the drug combination in vivo, as some of the EGR1-driven processes (invasion, angiogenesis, etc.) are especially relevant in the context of an organism [73].

### 3.6. An IMPDH Inhibitor Enhances the Potency of a MEK Inhibitor Against Uveal Melanoma In Vivo

**Figure 8:**
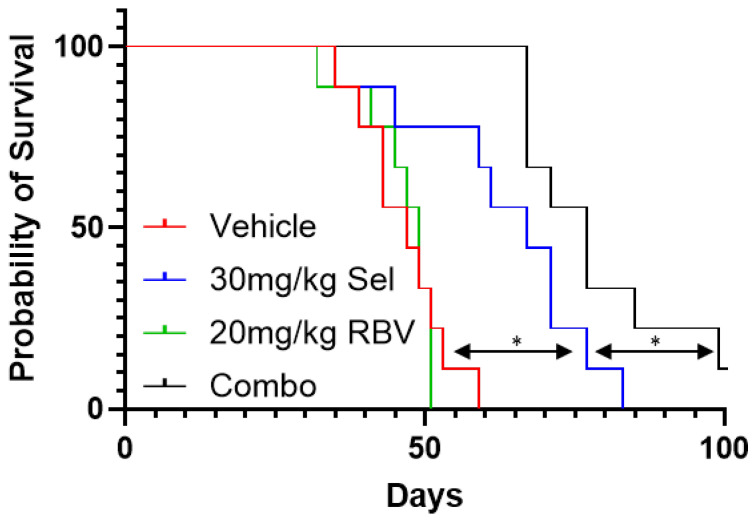
Ribavirin enhances the potency of selumetinib against uveal melanoma xenografts. The overall survival was determined in SCID mice harboring 92.1 xenografts and treated with 30 mg/kg selumetinib (“Sel”), 20mg/kg ribavirin (“RBV”), a combination thereof (“Combo”), or the vehicle alone (25% DMSO/30% PEG-300/45% water; “Vehicle”). The female mice inoculated with 92.1 cells were randomly assigned to the treatment groups, 9 mice per group, for daily oral gavage after the tumors reached ~100mm^3^. “*”—significantly different overall survival (*p* < 0.05; logrank test).

In order to elucidate whether the potentiation of MEK inhibitors by inhibitors of IMPDH, which we observed in vitro, persists in an organismal setting, we evaluated the efficacy of this combination in a xenograft tumor model. The 92.1 uveal melanoma cell line has been extensively used for subcutaneous xenograft studies in mice, providing a convenient model to evaluate the efficacy of potential therapeutic interventions [79]. As expected, treatment with selumetinib alone was able to extend the overall survival of the mice that carried 92.1-derived tumors (Figure 8). While the effect of ribavirin alone was insignificant, the combination of ribavirin and selumetinib was significantly more effective than selumetinib alone in prolonging the survival of the animals (Figure 8). No adverse effects attributable to the treatment were noticed in this experiment. Overall, these observations suggest that the addition of ribavirin to MEK inhibitor-based treatment regimens may enhance their potency against uveal melanoma in the context of an organism.

## 4. Discussion

Our observations suggest that targeting of the PAK-dependent signaling events enhances the efficacy of MEK inhibitors against UM. In this regard, GNAQ/GNA11-driven UM is similar to cutaneous melanomas that are driven by other oncogenes [19,20,21]. Direct targeting of PAK or RAC proteins, however desirable, remains currently out of reach for human patients. Despite the sustained effort by academic scientists and pharmaceutical companies [56,80,81], suitable inhibitors have not made it into clinical practice yet. For example, the pan-PAK ATP-competitive kinase inhibitor PF3758309 was withdrawn from phase 1 clinical trial in solid malignancies (clinicaltrials.gov ID NCT00932126) when it became apparent that it would not be sufficiently active at safely achievable doses. Importantly, our results indicate that PAK inhibitors may synergize with those of MEK. It is thus conceivable that otherwise ineffectual doses of PAK inhibitors might offer a meaningful clinical benefit as a part of a combination regimen. Reduction in the activity of, at least, group 1 PAKs can also be achieved indirectly, by preventing their activation by Rac-family GTPases [55]. One way to attain this is by preventing the interaction of a PAK protein with its upstream activators, as achieved by IPA3 [55]. Another approach would be to target RAC proteins directly. Accordingly, RAC inhibitors would be expected to have similar effects to those of PAK inhibitors. Indeed, in our experiments (Figure 3) we observed significant synergy between a MEK inhibitor selumetinib and a RAC inhibitor EHT1864, which prevents RAC1 from binding to nucleotides and activating PAK1 [60]. However, we are not aware of any direct RAC inhibitor currently approved for human use. Similarly, despite promising preclinical effects of liposomally formulated IPA3 [82], that agent is not available for human use at this time. Nevertheless, heightened dependence of RAC1 on locally produced GTP can be exploited via IMPDH inhibition [63], which gives an opportunity to control the pathway via clinically available IMPDH inhibitors. The synergistic relationship between the inhibitors of IMPDH with those of the MAPK cascade means that, for this combination as well, a significant effect can be achieved even when neither individual component is very efficacious on its own. Importantly, the availability of drugs such as ribavirin for human use opens up a possibility for clinical testing of IMPDHi/MEKi combinations while PAK and RAC inhibitors are still being developed or await regulatory approval.

Biochemically, the combination treatments achieve a better control of the MAPK cascade (Figure 5). This observation is consistent with the reports on PAK1 as a co-activator of CRAF and MEK1 [22,23,24,25], as well as on the dependence of GNAQ/GNA11 signaling on RAS and RAC proteins [30,31,32]. Indeed, the sensitivity of UM cells to PAK inhibition (Figure 1) is reminiscent of that of the cells driven by RAS oncogenes [20,26,27,57]. Notably, suppression of UM cells was seen in the range of doses that by themselves are essentially non-toxic to cancers driven by activated BRAF [20] and may even be protective to non-cancerous cells under certain stress conditions [58]. Improved control of the MAPK cascade by the combination treatment in our experiments is suggested by reduced phosphorylation of ERKs (Figure 5), as well as by reduced expression of the EGR1 gene (Figure 7B), which is a known transcriptional target of that signaling pathway [73]. Considering the reported biological functions of EGR1 [73], its downregulation may be expected to reduce proliferation, attenuate angiogenesis, and decrease invasiveness and metastasis. Of course, EGR1 is not the sole downstream target of ERKs. Furthermore, PAK1 mediates multiple additional pathways that affect growth, survival and therapeutic resistance in cancer cells [34,61]. Overall, the relative contribution of different affected effectors and pathways to the efficacy of the combination treatment remains to be elucidated.

The cell lines used in our study carry similar, but not identical, oncogenic drivers: GNAQ^Q209L^ in 92.1, GNAQ^Q209L/R210K^ in Mel202 and GNA11^Q209L^ in MP41. Predictably, they also differ in the mutations affecting other genes. For example, 92.1 harbors a G6D mutation in EIF1AX, while Mel202 carries CDKN2A^L65R^ and SF3B1^R625G^. It is encouraging to see that, despite those differences, the synergy between MEK inhibitors and the inhibitors of the RAC/PAK axis was observed in all the tested cell lines. These observations suggest that the phenomena observed in our study may be applicable to a broad range of uveal melanoma cases. However, as with any targeted therapy, it is reasonable to expect that the overall treatment outcome would depend on specific genetic and epigenetic peculiarities of each individual tumor. Importantly, PAK activity plays an important role in multiple resistance pathways that other cancers utilize to circumvent various targeted and conventional therapies [34]. It is thus possible that a regimen that includes PAK inhibitors would leave less room for the emergence of drug resistance. Nevertheless, further research is required to identify the possible determinants of resistance and sensitivity to the drug combinations discussed in the present study.

The specific dependence of RAC1 on de novo GTP production makes its activity vulnerable to conditions that have only a modest effect on the overall amounts of cellular GTP [62,63]. Therefore, in our experiments, we relied on moderate doses of IMPDH inhibitors. Importantly, RAC1 is more sensitive to partial GTP depletion than a number of other GTPases tested [62], while IMPDH inhibitors recapitulate direct inhibitors of RAC and PAK in synergistic drug combinations. However, we cannot formally rule out that there exist some unrelated cellular factors with similarly high sensitivity to fluctuations in the abundance of GTP. While such effects do not preclude safe use of ribavirin in the clinic or its efficacy in our experiments, they, theoretically, might contribute to the therapeutic activity of the drug combinations seen in our work. Importantly, if such additional anti-cancer effects indeed exist, they might further reduce the likelihood of complete insensitivity to such a therapy.

Importantly, the enzymes targeted for inhibition by the drugs in our study (RAC, PAK, IMPDH, MEK) are often represented by multiple isoforms. These isoforms, generally, have distinct, albeit often overlapping, expression patterns and biological activities. A remaining open question of considerable significance is whether future therapeutic intervention should focus on selective targeting of individual isoforms or strive to achieve the inhibition of multiple isoforms at once. By affecting only the most pertinent isoforms, the first approach may help reduce the adverse sequelae of the treatment, while the second approach might yield a higher overall efficacy and reduce the emergence of compensatory resistance mechanisms. The relative significance of individual isoforms and the possibility of cross-isoform compensation in the phenomena observed in our study still remain to be established.

While chemotherapy is traditionally aimed primarily at metastatic disease, the peculiarities of UM suggest a possible role for chemotherapeutic management of the localized form of this disease as well. The loss of an eye upon enucleation surgery is a serious quality-of-life issue, and even non-surgical methods of managing the primary UM are associated with a considerable risk of vision loss. On the other hand, the drug combinations described herein might suffice to delay progression of the disease enough for it not to be life-limiting. In this context, it is particularly notable that the targets of this combination (the MAPK cascade and the RAC/PAK axis) are generally known drivers of metastasis [61,83], and that the combined treatment is potent at suppressing expression of EGR1, which is specifically associated with metastasis and progression of UM ([78] and Figure 7A). An additional foreseeable benefit of enhanced suppression of the MAPK cascade may be in improving the tumor microenvironment, as this pathway shapes tumor-promoting stroma [84].

Of note, the concentrations of ribavirin used in our experiments are similar to what is encountered in human patients safely treated with this medication [85,86,87]. Initial in vitro experiments (Figure S3) suggested that an IMPDHi/MEKi combination, which is effective against uveal melanoma, may be tolerated by, at least, some non-transformed cells. This was further supported by the fact that the drug combination offered an overall survival benefit in vivo (Figure 8), suggesting the existence of a safe therapeutic window. It is possible that the outcomes can be further improved through dose escalation: the risks of side effects that currently limit the dose of ribavirin as an anti-viral agent might be more acceptable while treating an otherwise incurable malignancy.

Another possible direction for improving the efficacy of the IMPDHi/MEKi regimen is via combination with immunotherapy. Importantly, our in vivo observations were made in an immunocompromised animal model, and the interaction of the novel drug combinations with the immune system remains to be explored. Nevertheless, it is worth mentioning that ribavirin, a commonly used antiviral agent, at clinically active doses is not an immunosuppressant. In fact, RBV stimulates a pathway of T-cell differentiation, which may be advantageous for anti-viral and anti-cancer therapies [88,89]. Moreover, the drug combinations tested in our study demonstrated improved control of the MAPK cascade, while abundant evidence from various models suggest that inhibition of this pathway may be beneficial for immunotherapy [90,91,92]. Further research is warranted to establish whether additional benefit may be derived from supplementing various forms of immunotherapy with the drug combinations identified in this report.

## 5. Conclusions

In the present work, we have discovered that the activity of various MEK inhibitors against uveal melanoma cells in vitro and in vivo is significantly enhanced by concomitant inhibition of either RAC, PAK or IMPDH proteins. Some IMPDH inhibitors, including the widely used antiviral drug ribavirin, are readily available for human use, hence opening up a possibility for future clinical translation of our findings.

## Data Availability

The raw data supporting the conclusions of this article will be made available by the authors on request.

## Supplementary Materials

The following supporting information can be downloaded at: https://www.mdpi.com/article/10.3390/biom15101425/s1, Figure S1: Inhibitors of guanylate biosynthesis decrease GTP levels in uveal melanoma cells; Figure S2: Treatment with inhibitors of guanylate biosynthesis causes a decrease in the levels of active RAC1 in UM cells.; Figure S3: The response of non-transformed human cells to MEKi and IMPDHi treatment. Figure S4: IMPDHi ribavirin enhances the suppression of the MAPK cascade by MEKi selumetinib in Mel202 uveal melanoma cells.

## Abbreviations

The following abbreviations are used in this manuscript:

BIN	Binimetinib.
COB	Cobimetinib.
EHT	EHT1864.
GPCR	G-protein coupled receptor.
IMPDHi	Inhibitor of inosine monophosphate dehydrogenase (IMPDH).
MAPK	Mitogen-activated protein kinase
MEKi	Inhibitor of mitogen-activated protein kinase kinase (MEK).
MIR	Mirdametinib
MIZ	Mizoribine.
PAKi	Inhibitor of p21-activated protein kinase (PAK).
PF	PF-3758309.
RACi	Inhibitor of Ras-related C3 botulinum toxin substrate (RAC).
RBV	Ribavirin.
SEL	Selumetinib.
UM	Uveal melanoma
