# Supplementary material for: Inhibition of the RAC/PAK Signaling Axis Enhances the Potency of MAPK Cascade Inhibitors Against Uveal Melanoma
Source: Biomolecules. 2025 Oct 7;15(10):1425. doi: 10.3390/biom15101425 (PMC12564536; doi:10.3390/biom15101425)
Supplement: Supplementary file 1 [file biomolecules-15-01425-s001.zip › Suppl figs with original images.pdf]

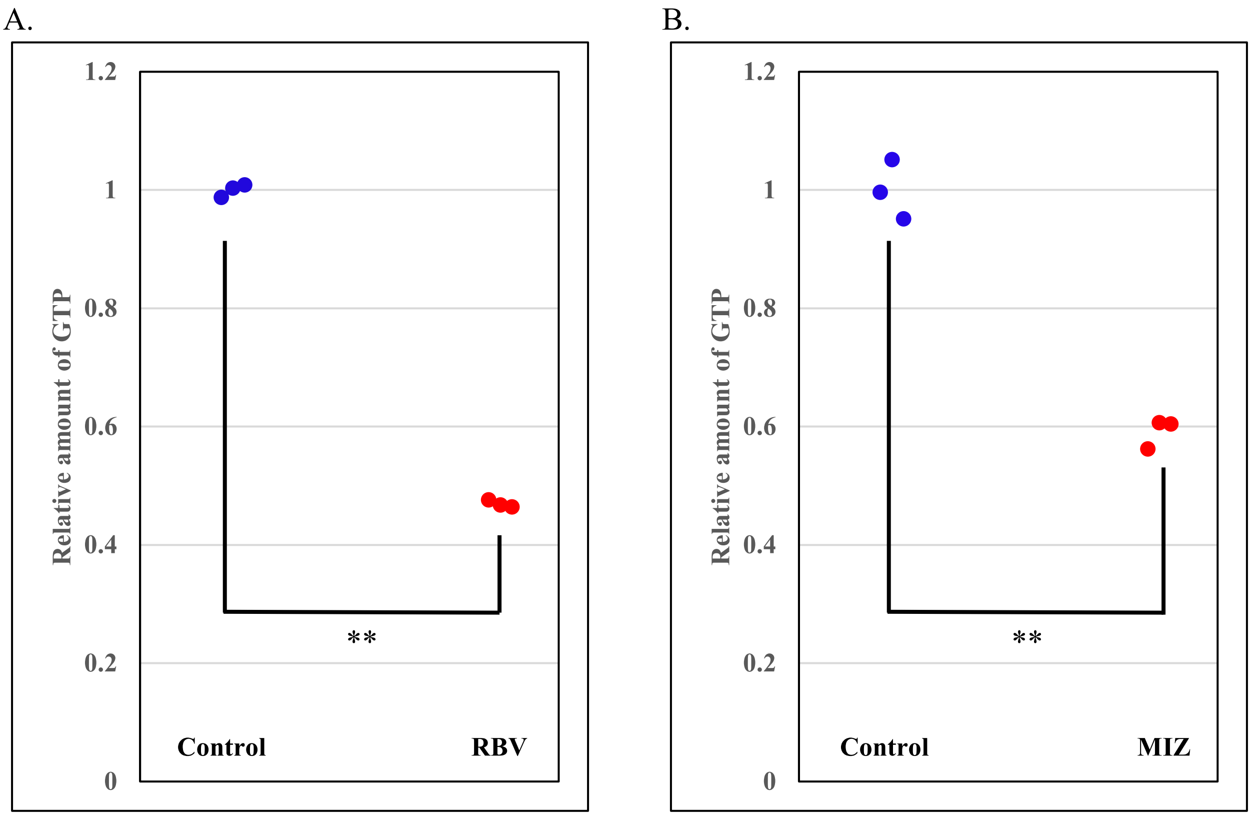

Figure S1. Inhibitors of guanylate biosynthesis decrease GTP levels in uveal melanoma cells. 92.1 uveal melanoma cells were treated with (**A**) 10µM ribavirin ("RBV") or (**B**) 10µM mizoribine ("MIZ") for 72 hours, and the levels of GTP were compared to those in parallel untreated control cultures. The levels of GTP are shown relative to the average values in the untreated cells. Colored dots represent biological replicas. "**" – statistically significant difference ($p < 0.005$; Student's t-test).

A.

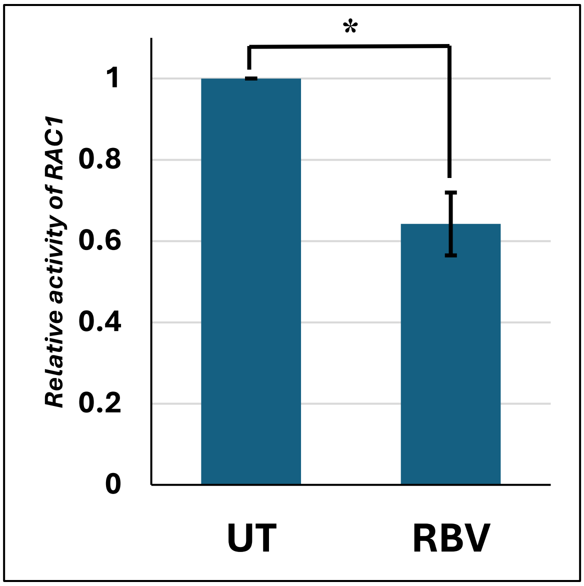

B.

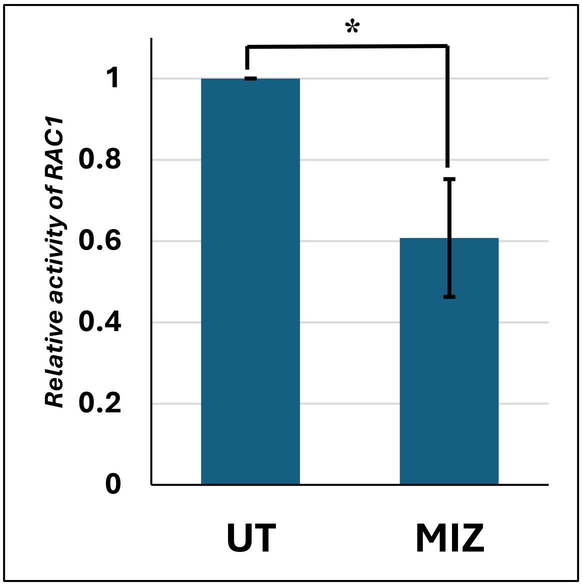

Figure S2. Treatment with inhibitors of guanylate biosynthesis causes a decrease in the levels of active RAC1 in UM cells. A. The amounts of active (capable of binding to PAK1) and total RAC1 were determined using the Active GTPase Kit (Cell Signaling Technology) in the lysates of 92.1 cells, untreated ("UT") or treated for 72 hours with 10µM ribavirin ("RBV"). The signals were acquired using Bio-Rad ChemiDoc Touch Imaging System and quantified using Fiji software (ImageJ version 1.54P). The amount of active RAC1 in a lysate was normalized to that of the total RAC1 protein and is shown relatively to the value in the parallel untreated culture in the same experiment. The average values from five independent experiments are shown. Error bars denote the standard errors of the mean. "*" – $p < 0.05$ (Student's t-test). **B.** Relative activity of RAC1 in 92.1 cells, untreated ("UT") or treated for 72 with 10µM mizoribine ("MIZ"), was determined and analyzed as in A. The average values from four independent experiments are shown. Error bars denote standard errors. "*" – $p < 0.05$ (Student's t-test).

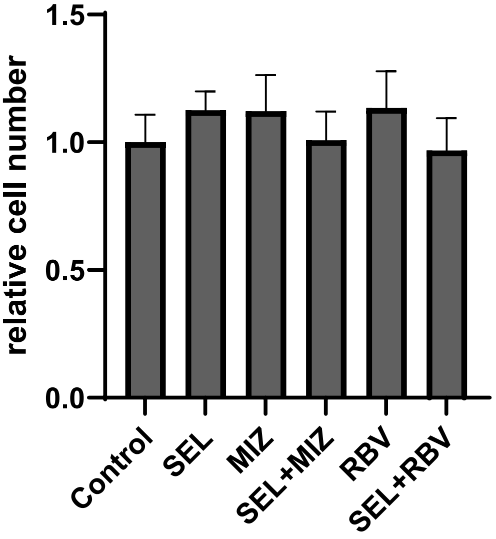

Figure S3. The response of non-transformed human cells to MEKi and IMPDHi treatment. Human normal fibroblasts immortalized by telomerase expression (WI-38-TERT) were treated for 5 days with 30nM selumetinib ("SEL"), 6μM mizoribine ("MIZ"), selumetinib and mizoribine combined ("SEL+MIZ"), 12μM ribavirin ("RBV"), or selumetinib and ribavirin combined ("SEL+RBV"). The numbers of the remaining cells were compared using the methylene blue staining and extraction method and are shown relative to those in untreated control populations. Error bars denote 95% confidence intervals. The differences between the control cells and each of the drug-treated populations are statistically insignificant (p>>0.05; ANOVA with Dunnet's test).

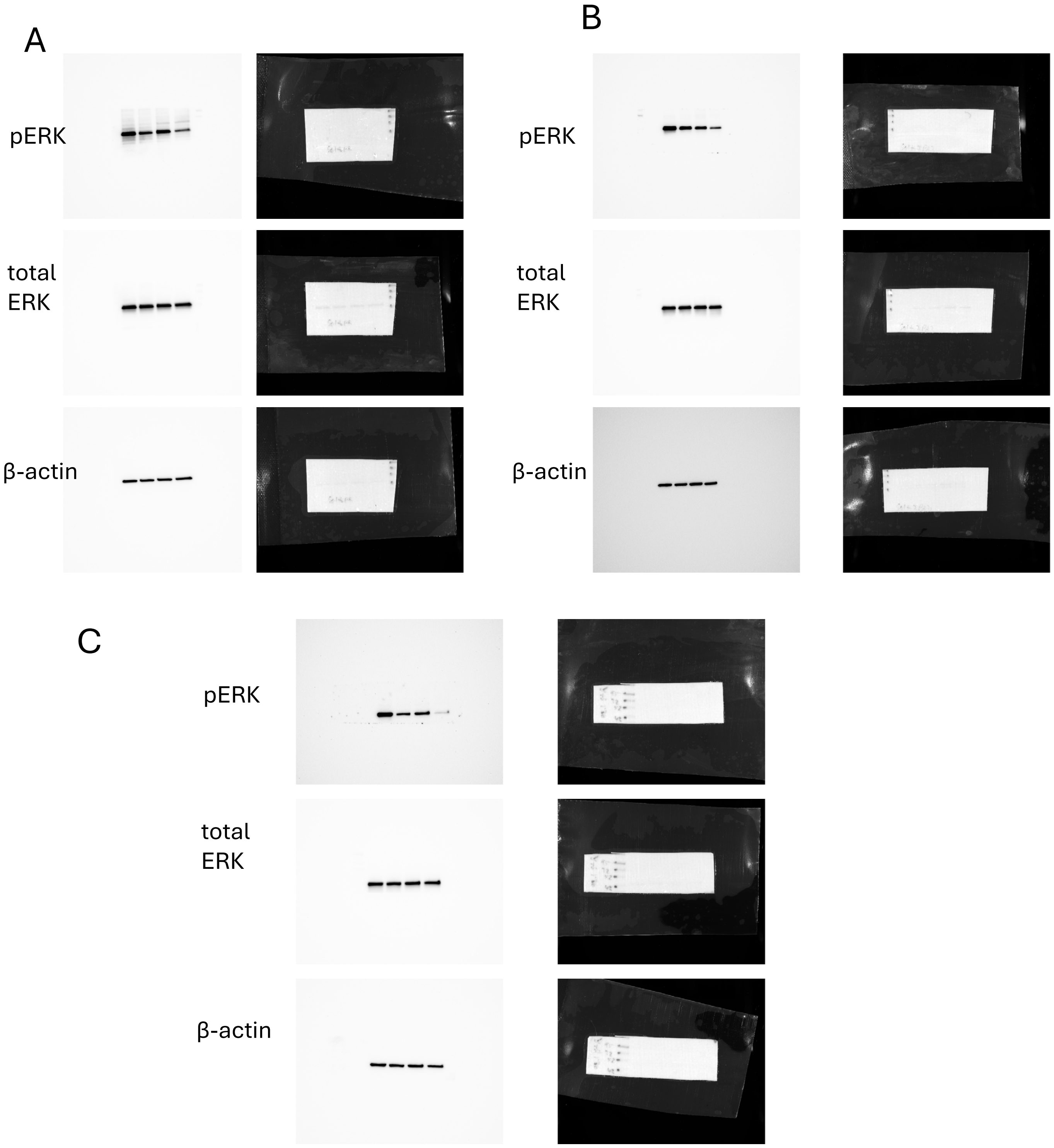

Figure S4. The original Western blotting images for Figure 5.

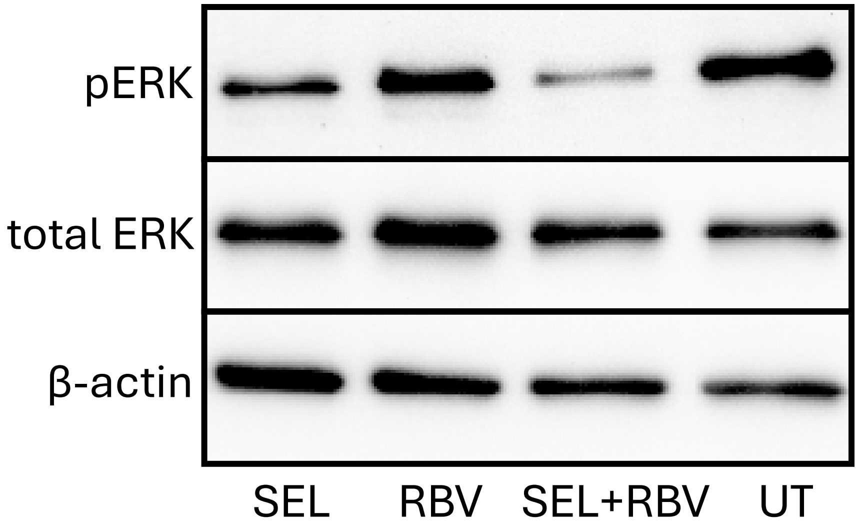

Figure S5. IMPDHi ribavirin enhances the suppression of the MAPK cascade by MEKi selumetinib in Mel202 uveal melanoma cells. Lysates of 92.1 cells treated for 72h with selumetinib (30nM; “SEL”), ribavirin (10µM; “RBV”) or their combination (“SEL+RBV”) were compared to those of untreated cultures (“UT”; exposed only to the vehicle DMSO) by probing with the antibodies for phosphorylated ERK (top), total ERK (middle) and β-actin (bottom).

pERK

total
ERK

β-actin

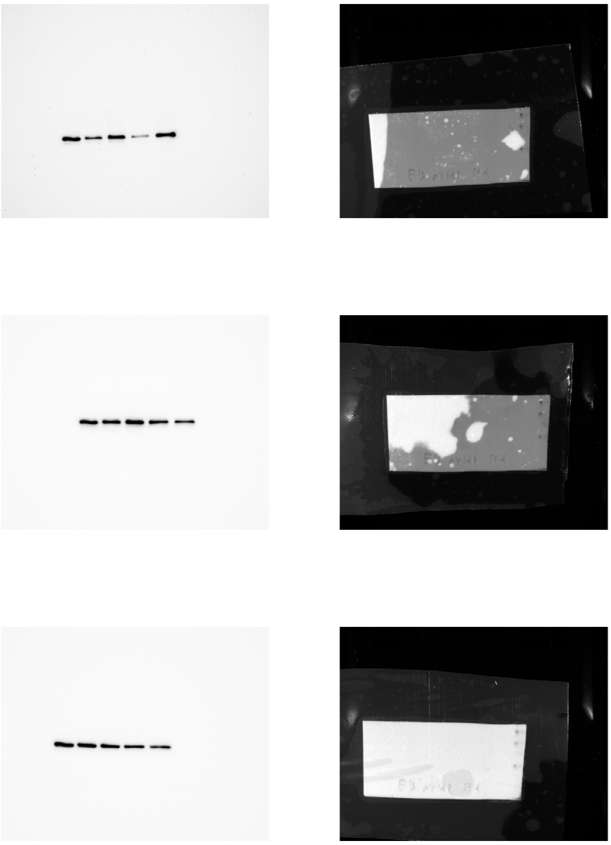

Figure S6. The original Western blotting images for Figure S5.
